# Liver abscess caused by *Clostridium haemolyticum* infection after transarterial chemoembolization for hepatocellular carcinoma: A case report

**DOI:** 10.1097/MD.0000000000010688

**Published:** 2018-05-11

**Authors:** Dong-Jun Son, Ji-Yun Hong, Ki-Hyun Kim, Young-Hoon Jeong, Dae-Seong Myung, Sung-Bum Cho, Wan-Sik Lee, Yang-Jun Kang, Jin-Woong Kim, Young-Eun Joo

**Affiliations:** a Department of Internal Medicine, Chonnam National University Medical School; b Department of Radiology, Chonnam National University Medical School, Gwangju, Korea.

**Keywords:** *Clostridium*, hepatocellular carcinoma, liver abscess, transarterial chemoembolization

## Abstract

**Rationale:**

Liver abscesses caused by *Clostridium* species infection are extremely rare.

**Patient concerns:**

The authors report the first case of a liver abscess due to *Clostridium haemolyticum*, which occurred after transarterial chemoembolization (TACE) for hepatocellular carcinoma, in a 76-year-old woman who presented with right upper quadrant pain and fever.

**Diagnoses:**

Computed tomography of the abdomen after the second TACE showed an air-filled abscess around a compact, lipiodolized lesion in the right hepatic lobe. Pus culture showed the growth of *C haemolyticum*.

**Interventions:**

Broad-spectrum antibiotics, including piperacillin/tazobactam and metronidazole, were administered, and a percutaneous 10-French pigtail catheter for pus drainage and culture was inserted in the liver abscess.

**Outcomes:**

Despite administering intensive treatments, she presented with rapid deterioration in mental status, liver function, and infection markers. She was transferred to the local hospital for palliative conservative treatment.

**Lessons:**

Clostridia infections, including those involving *C haemolyticum*, are extremely rare, but should be considered as one of the causative organisms of liver abscess formation after TACE because of its rapid and fatal clinical course.

## 1 Introduction

Hepatocellular carcinoma (HCC) is one of the most common cancers and is still one of the major causes of cancer-related morbidity and mortality worldwide. Recent advances in treatment modalities, such as surgical resection, liver transplantation, radiofrequency ablation, and transarterial chemoembolization (TACE), have prolonged the survival of patients with HCC.^[1,2]^

TACE is the most widely used primary treatment for unresectable HCC and metastatic liver cancer, and is generally well-tolerated. However, previous studies reported major complications associated with TACE including hepatic failure, hepatic rupture, liver infarction, and liver abscesses.^[3–5]^

The incidence rate of liver abscess formation after TACE is low, but is one of the serious complications with a high mortality rate. The causative organisms of liver abscesses are frequently enteric in origin, such as *Escherichia coli*, *Enterobacter cloacae*, and *Enterococcus faecalis*.^[6–11]^ Until now, few cases of liver abscesses derived from anaerobic bacilli of the *Clostridium* species, which are inhabitants of the human intestinal and genital tract, after TACE have been reported. The causative organisms were *Clostridium perfringens* and *Clostridium difficile*.^[12–16]^ To our knowledge, liver abscesses derived from *Clostridium haemolyticum* infection after TACE have never been reported.

Here, we present a case of a liver abscess caused by *C haemolyticum* infection after TACE for HCC and review the literatures pertaining to this condition.

An ethics committee approved the study. Informed consent was given to the patient.

## 2 Case report

**Figure 1:**
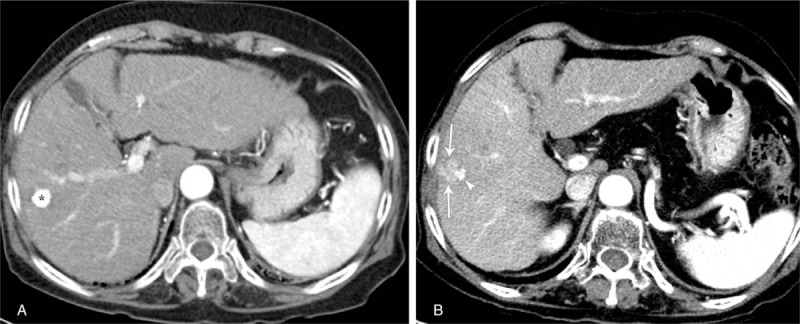
(A) Contrast-enhanced axial arterial phase CT image shows a compact lipiodolized lesion (asterisk) treated with TACE for a small HCC in the right hepatic lobe. (B) Fourteen-month follow-up contrast-enhanced axial arterial phase CT image depicts arterial enhancement in a recurrent viable HCC (arrows) around a contracted lipiodolized lesion (arrowhead) in the right hepatic lobe. CT = computed tomography, HCC = hepatocellular carcinoma, TACE = transarterial chemoembolization.

**Figure 2:**
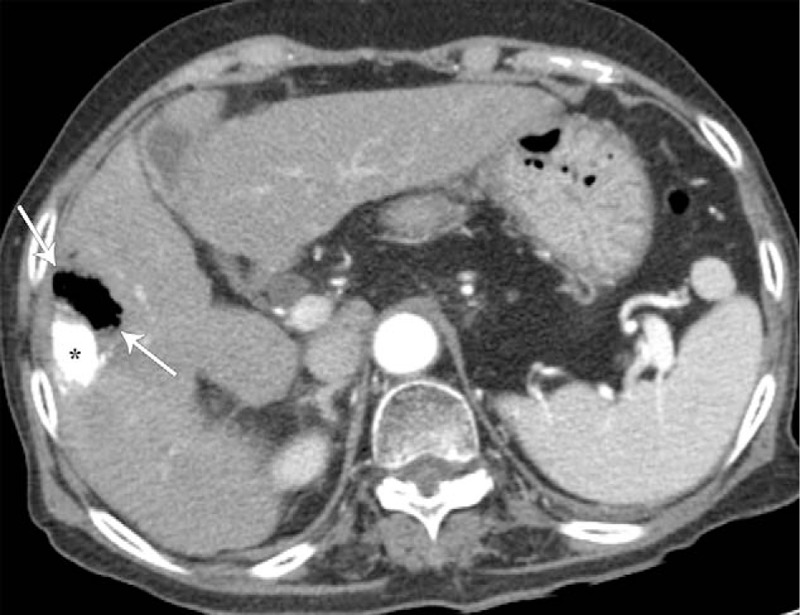
Two-week follow-up contrast-enhanced axial CT image after second TACE for a recurrent HCC shows an air-filled abscess (arrows) around a compact lipiodolized lesion (asterisk) in the right hepatic lobe. CT = computed tomography, HCC = hepatocellular carcinoma, TACE = transarterial chemoembolization.

**Figure 3:**
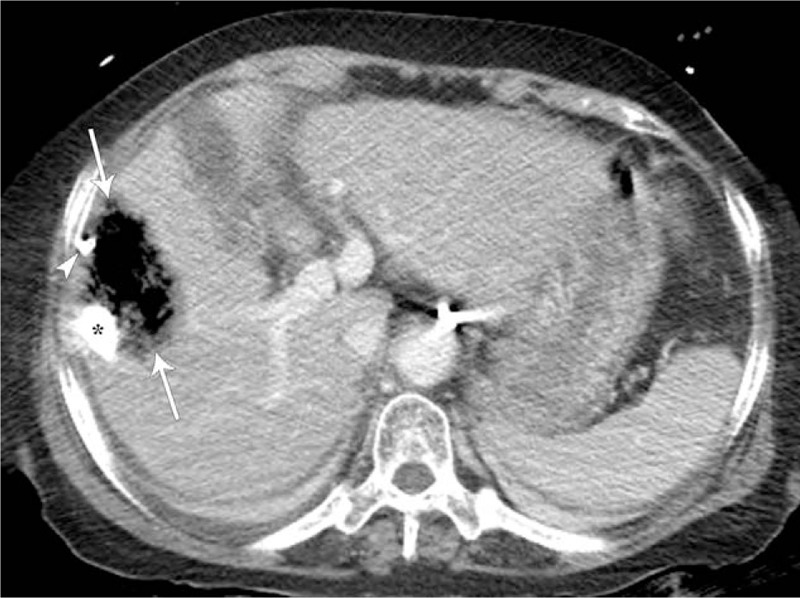
Three-week follow-up contrast-enhanced axial CT image after second TACE demonstrates a pigtail catheter (arrowhead) for percutaneous drainage of an aggravated air-filled abscess (arrows) around a compact lipiodolized lesion (asterisk) in the right hepatic lobe. CT = computed tomography, TACE = transarterial chemoembolization.

**Figure 4:**
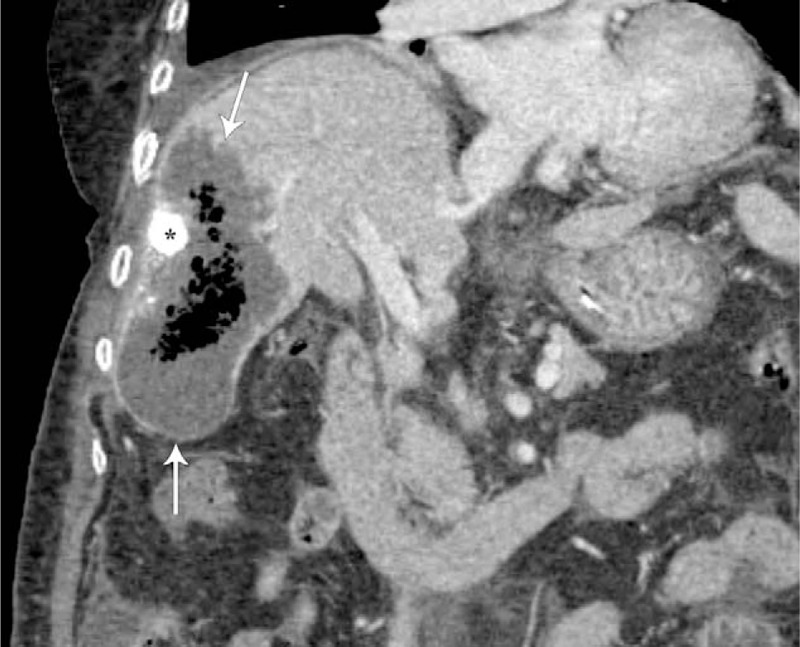
Five-week follow-up contrast-enhanced coronal CT image shows a more aggravated air-filled abscess (arrows) around a compact lipiodolized lesion (asterisk) in the right hepatic lobe, despite percutaneous drainage. CT = computed tomography.

A 76-year-old woman was admitted to our hospital for her second TACE for HCC. She had suffered from HCC due to chronic hepatitis B for 2 years and had undergone her first TACE at another hospital. She has been receiving entecavir for chronic hepatitis B for 10 years. She also has diabetes mellitus and hypertension. Computed tomography (CT) of the abdomen after her first TACE showed a compact lipiodolized lesion treated with TACE for a small HCC in the right hepatic lobe (Fig. 1A) and a 14-month follow-up CT showed a recurrent viable HCC with arterial enhancement around a contracted lipiodolized lesion in the right hepatic lobe (Fig. 1B). On admission, laboratory examinations revealed the following: white blood cell count, 4000/mm^3^ (normal range, 6000–10,000/mm^3^); hemoglobin, 13.5 g/dL (normal range, 12–16 g/dL); platelet count, 94,000/mm^3^ (normal range, 130,000–450,000/mm^3^); serum albumin, 4.1 g/dL (normal range, 3.0–5.0 g/dL); aspartate aminotransferase, 19 U/L (normal range, 5–37 U/L); alanine aminotransferase, 15 U/L (normal range, 5–40 U/L); alkaline phosphatase, 113 U/L (normal range, 39–117 U/L); γ-glutamyl transpeptidase, 43 U/L (normal range, 7–49 U/L); total bilirubin, 0.51 mg/dL (normal range, 0.2–1.2 mg/dL) with 0.16 mg/dL direct fraction (normal range, 0.05–0.3 mg/dL); alpha-fetoprotein, 244 IU/mL (normal range, 0.74–7.29 IU/mL); and protein induced by vitamin K absence-2, 489 mAU/mL (normal range, 0–40 mAU/mL). We performed TACE with right hepatic arterial infusion of adriamycin (20 mg) and lipiodol (4 mL), followed by gelatin (250 μm) chemoembolization. After TACE, she did not exhibit abnormal physical and laboratory examination results and was discharged. Two weeks after TACE, she returned to our hospital with right upper quadrant pain with fever and she was drowsy. The abdomen was mildly distended, with tenderness in the right upper quadrant. Laboratory examinations showed liver dysfunction and infection as follows: white blood cell count, 5300/mm^3^; hemoglobin, 10.6 g/dL; platelet count, 40,000/mm^3^; serum albumin, 2.3 g/dL; aspartate aminotransferase, 303 U/L; alanine aminotransferase, 616 U/L; alkaline phosphatase, 113 U/L; γ-glutamyl transpeptidase, 110 U/L; total bilirubin, 3.0 mg/dL with 2.1 mg/dL direct fraction; ammonia, 55 (normal range, 12–66); lactate dehydrogenase, 1212 IU/L (normal range, 218–472 IU/L); and C-reactive protein, 32.16 mg/dL (normal range, 0–40 mg/dL). Follow-up CT of the abdomen after the second TACE showed an air-filled abscess around a compact, lipiodolized lesion in the right hepatic lobe (Fig. 2). Broad-spectrum antibiotics, including piperacillin/tazobactam and metronidazole, were administered after collecting blood cultures, and a percutaneous 10-French pigtail catheter for pus drainage and culture was inserted in the liver abscess. She was admitted to the intensive care unit for close observation. Pus culture later showed the growth of gram-positive bacilli, which were identified as *C haemolyticum*. No microorganism was found in blood and urine cultures. Antibiotics were changed to tigecycline, which was proven to be sensitive to *C haemolyticum*. Seven days after percutaneous drainage catheter insertion, laboratory examinations showed improvement of liver dysfunction and infection markers, and follow-up CT of the abdomen showed a pigtail catheter for percutaneous drainage of an aggravated air-filled abscess around a compact lipiodolized lesion in the right hepatic lobe (Fig. 3). However, her mental status showed a tendency to worsen without improvement. Brain magnetic resonance (MR) imaging with MR angiography and diffusion revealed multifocal nodular and patchy T2 hyperintense lesions with diffusion restriction and some nodular and peripheral enhancement in both cerebral hemispheres, both basal ganglia, left thalamus, both temporo-occipital lobes, and both cerebellar hemispheres, suggestive of invasive fungal infection. A brain biopsy and/or spinal tapping for cerebrospinal fluid examination for accurate diagnosis were needed. However, her family refused any further invasive examinations. Intravenous amphotericin B, an anti-fungal agent, was added empirically. Fourteen days after admission, follow-up CT of the abdomen showed a more aggravated air-filled abscess around a compact lipiodolized lesion in the right hepatic lobe, despite percutaneous drainage (Fig. 4). Despite administering intensive treatments, she presented with rapid deterioration in mental status, liver function, and infection markers. She was transferred to the local hospital for palliative conservative treatment.

## 3 Discussion

The normal liver has a dual blood supply from the portal vein and hepatic artery, while HCC is mainly dependent on the hepatic artery for blood supply.^[1,2]^ TACE is performed by embolization of the blood supply of the HCC via super-selective catheterization of the hepatic artery and the targeted delivery of chemotherapeutic agents to the HCC.^[3–5]^ Although it is recognized as a safe treatment, various complications such as self-limiting postembolization syndrome, biloma, bacteremia, hepatic failure, cholecystitis, gastrointestinal bleeding, pancreatitis, renal failure, liver infarction, and liver abscesses have been reported.^[3–5]^ The complications after TACE are usually caused by inadvertent embolization, portal vein obstruction, previous gastrointestinal or biliary surgery, biliary obstruction, large tumor size, deterioration of reserve liver function, concomitant diabetes mellitus, and cytotoxicity by chemotherapeutic agents.^[3–5]^

The incidence rate of liver abscesses after TACE is relatively low, ranging from 0.1% to 4.5%, but it is a serious complication with significant morbidity and mortality rates.^[6–11]^ The mechanisms of liver abscess formation after TACE remain unclear. Previous reports showed that the most common predisposing risk factor was a history of bilioenteric anastomosis, endoscopic sphincterotomy and stenting, or biliary external drainage.^[10,11]^ Therefore, liver abscess formation may be attributed to liquefactive necrosis of tumors after TACE and bacterial infection through the biliary tree and gastrointestinal tract into the necrotic tumor. However, in our case, the patient did not undergo gastrointestinal or biliary tract surgery or procedures.

Although liver abscesses after TACE are associated with high morbidity and mortality rates, until now, prophylactic antibiotic administration pre- and peri-procedure have not been recommended routinely, even in high-risk patients such as those with a history of bilioenteric anastomosis, endoscopic sphincterotomy and stenting, or biliary external drainage, because the overall rate of liver abscesses after TACE is low.^[6–11]^

The most common bacterium of liver abscesses is *E coli*, followed by *E cloacae*, *E faecalis*, *Klebsiella pneumonia*, and then *Staphylococcus epidermidis*.^[6–11]^*Clostridium* species are the gram-positive, gas-forming, and rod-shaped anaerobic bacilli, which are normal inhabitants of the human gastrointestinal and genital tracts. Clostridia infections are rare and are often associated with old age, liver cirrhosis, diabetes mellitus, malignancies, immunocompromised state, and invasive procedures such as TACE.^[12–16]^*Clostridium* liver abscesses after TACE are extremely rare. Until now, few cases of *Clostridium* liver abscesses associated with TACE have been documented in the literature.^[12–16]^ In the reported cases of *Clostridium* liver abscesses, the pathogens were mostly *C perfringens* or *C difficile*.^[12–16]^ However, *C haemolyticum* as the causative organism of liver abscesses has never been reported. To our knowledge, this is the first report on a liver abscess caused by *C haemolyticum* after TACE.

The clinical courses of reported cases of *Clostridium* liver abscesses were mostly rapid and fatal.^[12–16]^ Therefore, *Clostridium* liver abscesses require prompt treatment. Effective treatment of these abscesses relies on administration of sensitive antibiotics in a timely manner and prompt percutaneous drainage of abscess.^[17,18]^ In our case, broad-spectrum antibiotics for coverage of gram-positive and gram-negative aerobic and anaerobic organisms were administered after collecting blood cultures and a percutaneous catheter for pus drainage and culture was inserted promptly. All blood, urine, and pus cultures with antibiotic sensitivity tests were performed. Positive microbiological isolates were obtained in pus culture. According to results of the antibiotic sensitivity test, antibiotics were switched to another, which may have been sensitive to *C haemolyticum*.

Percutaneous drainage is a safe and effective method for treating liver abscesses following TACE.^[6–11]^ In our case, percutaneous drainage was performed promptly. However, sensitive antibiotics and percutaneous drainage did not adequately control the liver abscess caused by *C haemolyticum* after TACE in our case without other risk factors except old age, diabetes mellitus, and underlying liver cirrhosis.

In conclusion, liver abscess formation after TACE is rare, but a serious complication with significant morbidity and mortality. Clostridia infections, including those involving *C haemolyticum*, are extremely rare, but should be considered as one of the causative organisms of liver abscess formation after TACE because of its rapid and fatal clinical course. Broad-spectrum antibiotics and percutaneous drainage are still a first-line management for liver abscesses following TACE, although they were not effective in our case.

## Author contributions

**Conceptualization:** Dae-Seong Myung, Sung-Bum Cho, Young-Eun Joo.

**Data curation:** Ki-Hyun Kim, Young-Hoon Jeong.

**Investigation:** Ji-Yun Hong.

**Resources:** Yang-Jun Kang, Jin-Woong Kim.

**Supervision:** Dae-Seong Myung, Sung-Bum Cho, Wan-Sik Lee, Young-Eun Joo.

**Writing – original draft:** Dong-Jun Son.

**Writing – review and editing:** Young-Eun Joo.
